# Conversion of vesicostomy into continent catheterizable reservoirs in myelomeningocele

**DOI:** 10.1590/S1677-5538.IBJU.2020.0385

**Published:** 2021-02-03

**Authors:** Antonio Macedo, Sérgio Leite Ottoni, Gilmar Garrone, Ricardo Marcondes Mattos, Marcela Leal da Cruz

**Affiliations:** 1 CACAU-NUPEP-AACD Departamento de Urologia São Paulo SP Brasil Departamento de Urologia, CACAU-NUPEP-AACD, São Paulo, SP, Brasil; 2 Universidade Federal de São Paulo Departamento de Pediatria São Paulo SP Brasil Departamento de Pediatria, Universidade Federal de São Paulo - UNIFESP, São Paulo, SP, Brasil

## Abstract

**Introduction:**

Vesicostomy should be considered in children with neuropathic bladder in case first-line therapies fail. This simple and reversible procedure can reduce febrile urinary tract infections and protect the upper urinary tract (1) until more definitive alternatives can be proposed.

We describe in this video how we approach patients that underwent vesicostomy and want it to be converted into a continent catheterizable reservoir.

**Material and methods:**

We perform an infra-umbilical longitudinal incision with a semicircular flap where the stoma will be placed (outside vesicostomy). After releasing the bladder, we proceed with usual steps of the Macedo-Pouch technique (2). We perform the reservoir from 35cm of ileum that constructs a catheterizable channel from the same bowel segment from a 3cm width flap from anterior and posterior wall of ileum in the mid part of it. The continence mechanism of the efferent tube is based on angulation and a serous lined tunnel created with 3-4 3.0 prolene sutures. The stoma is placed in the midline (3).

**Results:**

Patient had an uneventful evolution and is continent performing CIC every 4 hours with 9 months of follow-up. We have in the last 3 years a consecutive series of 12 patients operated according to this principle.

**Discussion:**

Vesicostomy should be regarded as an alternative for patients with neurogenic bladder refractory to clinical treatment at a younger age in order to postpone definitive treatment such as any an enterocystoplasty.

This option must be considered as transient, since definitive reconstructive surgery can provide preservation of renal function and continence achievement. In this context, our video demonstrates that performing a bladder augmentation on a patient with a vesicostomy is safe and feasible. We reinforce that our method precludes the need of appendix or creation of a Monti tube as the outlet channel and the whole procedure is performed from a single piece of bowel.

Available at: http://www.intbrazjurol.com.br/video-section/20200385_Cruz_et_al Int Braz J Urol. 2021; 47 (Video #09): 470-1
